# The Effect of Different Rotary Systems on Creating Intracanal Dentinal Defects: An *Ex Vivo* Study 

**DOI:** 10.30476/dentjods.2025.103410.2449

**Published:** 2026-03-01

**Authors:** Abbas Abbaszadegan, Alireza Adl, Fateme Nemati, Nikta Ranjbar

**Affiliations:** 1 Dept. of Endodontics, School of Dentistry, Shiraz University of Medical Sciences, Shiraz, Iran.; 2 Dept. of Endodontics, Biomaterials Research Center, School of Dentistry, Shiraz University of Medical Sciences, Shiraz, Iran.; 3 Undergraduate Students, Shiraz University of Medical Sciences, Shiraz, Iran.; 4 Private Practice, Shiraz, Iran.

**Keywords:** Dentine, Root canal preparation, Instrumentation, Tooth fracture

## Abstract

**Background:**

The creation of intracanal dentinal defects during root canal preparation is a concern in endodontic treatment; as such defects can compromise the integrity of the tooth and potentially lead to fractures or failure of the treatment. Rotary systems, commonly used for shaping root canals, vary in their design, material, and cutting mechanisms, which may influence the extent of dentinal defects they induce. However, the comparative impact of different rotary systems on the formation of these defects remains unclear.

**Purpose:**

The aim of this *ex vivo* study was to compare the effects of four rotary systems (One-curve, One-shape, Neolix, and ProTaper Universal) on the creation of intracanal dentinal defects in extracted human teeth.

**Materials and Method:**

In this *ex vivo* study, seventy extracted human mandibular incisor teeth with straight roots and no extra canals or existing dentinal defects were selected and randomly divided into five groups, including one control group and four experimental groups. In the control group (n=10), no instrumentation was performed. In the experimental groups (n=15 each), instrumentation was done using the rotary systems ProTaper Universal, Neolix, One-shape, and One-curve, respectively. All groups received the same amount of irrigation: 12mL of 2% sodium hypochlorite followed by 3mL of sterile saline. The roots were then horizontally sectioned at 3, 6, and 9 mm from the apex and evaluated under a stereomicroscope for the presence of intracanal defects. Data were analyzed using Chi-square test.

**Results:**

The lowest and highest rates of dentinal cracks were observed in the One-curve and One-shape groups, respectively. No significant differences were observed among the experimental groups (*p* Value=0.46).

**Conclusion:**

All tested rotary systems induced dentinal defects. The lowest and highest incidence of dentinal defects occurred in teeth prepared using the One-curve (26%) and One-shape (53%) rotary instruments, respectively.

## Introduction

One of the most unfavorable consequences of endodontic treatments, which may arise during root canal preparation, is the creation of dentinal cracks and craze lines. These defects can lead to vertical root fractures, impacting the outcome of root canal therapy and the prognosis of treatment [ 1 ]. 

Several factors contribute to the formation of incomplete dentinal fractures, craze lines, and micro-cracks. These include the use of nickel-titanium (Ni-Ti) rotary instruments for root canal preparation, dentine dehydration, and irrigation solutions, particularly high-concentration sodium hypochlorite (NaOCl) [ 2 - 6 ]. 

Specific parameters of each rotary system may influence the formation of dentinal defects, such as blade design, the number of cutting edges, file taper, cross-sectional shape, and rake angle [ 2 , 5 ]. Rotary files create higher stress on dentin compared to hand files due to their greater taper and the increased rotations required inside the lumen [ 7-8 ]. 

Recently, single-file rotary systems have gained popularity due to their advantages, including reduced working time, lower cross-contamination, and improved safety during shaping procedures [ 9 ]. However, using only one file for the preparation process may increase the risk of dentinal defects due to stress concentration in the lumen [ 10 ]. 

The first rotary single-file system, One Shape (Micro Mega, France), was introduced to the dental market in 2011. It features a continuous clockwise motion and requires endodontic motors [ 11 ]. One Shape files have a safe, non-cutting tip and three different cross-sections along the active length: a triangular or modified triangular cross-section with three sharp cutting edges in the middle and apical thirds, and an S-shaped cross-section with two cutting edges near the shaft [ 11 - 12 ]. The One-Curve rotary system (Micro Mega, France), another single-file system from the same company introduced in 2018, also has varying cross-sectional designs along the shaft, enabling effective cutting ability [ 13 ]. 

Neolix (Neolix, Châtres-la-Forêt, France) is also a single-file rotary system [ 14 ] with continuous motion. These files have a non-cutting tip to prevent transportation and ensure safe instrumentation [ 15 ]. They are manufactured using a wire-cut electrical discharge machining process, which creates a rough surface with abrasive properties, resulting in faster root canal preparation. Additionally, they are heat-treated to enhance flexibility [ 16 ]. 

The ProTaper Universal system (Dentsply/Maillefer, Ballaigues, Switzerland) is a widely used rotary instrument made from conventional super-elastic NiTi wire. It has a convex triangular cross-sectional design with a progressive taper along the file length and an aggressive cutting action, which removes relatively more dentin coronally [ 17 ]. The Protaper system in commonly utilized across numerous countries and in included in the curricula of undergraduate dental programs. Furthermore, it has been extensively investigated in the literature, making it a suitable standard for use in the present study [ 3 , 18 ]. 

To the best of our knowledge, there are limited studies comparing the effects of different single-file rotary systems on the formation of dentinal defects. Therefore, this study was designed to assess the potential for dentinal defects induced by the ProTaper, One Shape, One Curve, and Neolix rotary systems.

## Materials and Method

This study received approval from the ethics committee of Shiraz University of Medical Sciences (IR.SUMS.DENTAL.REC.1399.086).

The sample size was calculated based on at least a moderate difference between the groups (effect size w=0.30), type I error rate α=0.05, and type II error rate β=0.20. Therefore, the sample size was calculated to be 15 teeth in each experimental group. Seventy extracted single-canal mandibular incisors were selected for this study. These teeth had been extracted as a result of orthodontic treatment or periodontal diseases. The selected teeth were washed, soft tissue and calculus were removed and were then stored in distilled water until use. Teeth with curved roots, calcifications, root decay, and other defects were excluded. To exclude teeth with external root cracks, all samples were evaluated using a stereomicroscope (BestScope, BS-3060C, China) with 20× magnification.

Teeth were radiographically examined in both mesiodistal and buccolingual directions to exclude any extra canals. All teeth were decoronated using a high-speed diamond bur (Tizkavan, Iran) with water spray to reach a standard root length of 13mm. Aluminum layers were wrapped around each tooth to mimic the periodontal area, and the teeth were mounted in silicone impression material (Heavy Body, Coltene, Germany) in square-shaped bottles. After setting, the impression materials were removed from the bottles, and the aluminum foils were taken out. Then, the free spaces were filled with light body silicone impression material (Coltene, Germany). Samples were randomly divided into four experimental groups (n=15 each) and one control group (n=10).In the control group, no instrumentation was performed; only irrigation was done. In ProTaper Universal group, files from S1 to F2 were used (S1, and S2, 300 rpm and 3 Ncm; F1 and F2, 300 rpm and 2 Ncm). In the single files groups, One Curve rotary file (25/06, 300 rpm, 2.5 Ncm), One Shape single rotary file (25/06, 400rpm, 4Ncm), and Neolix rotary system (25/06, 400rpm, 1.5Ncm) were used. In all groups, a manual glide path was established using K-files #10 and #15. An electronic motor (NSK, Endo-Mate DI, Model NE, Japan) was used for preparation according to the manufacturer’s instructions for each rotary system. Irrigation was done similarly for all groups using 12mL of 2% NaOCl (Cerkamed, Poland) followed by 3mL of sterile saline as final irrigation, using a #27 needle gauge (Ava, Iran). After root canal preparation, the roots were removed from their molds and horizontally sectioned at 3, 6, and 9mm from the apex using a saw (Mecatome T180, Presi SA, Angonnes, France) with water cooling. All the segments were soaked in methylene blue to increase the accuracy of crack line detection. The segments were then evaluated by two expert operators, who were blinded to the study, using a stereomicroscope with 25× magnification. If there was a disagreement between the two observers, they checked the samples together to reach an agreement.

 If in any section of a root, an incomplete crack originating from inside the canal or a complete crack was observed, that root was considered as "cracked" [ 19 ]. Statistical analysis was done using Chi-square with SPSS 23 (SPSS Inc, Chicago, Illinois, USA). Level of significance was set at 0.05.

## Results

**Figure 1:**
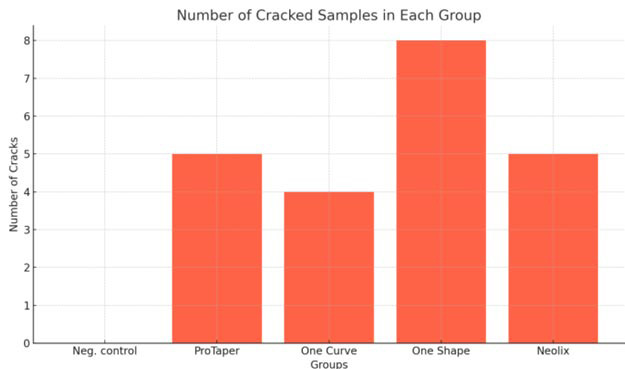
Distribution of cracks in different groups

The number and percentage of intra-canal cracks in different groups are shown in Table 1 and Figure 1. No cracks were found in the control group. Among the experimental groups, the lowest and highest rates of dentinal cracks were observed in the One-curve and One- shape groups, respectively. According to the Chi-square test, there was no significant difference in the number of dentinal cracks among the experimental groups (*p*= 0.46). 

**Table 1:** The number and percentage of intra-canal cracks in different groups

Groups	Number	+ crack	- crack	% crack
Neg. control	10	0	10	0
ProTaper	15	5	10	33
One-curve	15	4	11	26
One-shape	15	8	7	53
Neolix	15	5	10	33

## Discussion

An important drawback of NiTi rotary systems is the possibility of dentinal crack formation during root canal preparation, despite their advantages such as time-saving, flexibility, and reduced clinician fatigue [ 20 ]. This study aimed to compare four rotary systems (ProT-aper Universal, One-curve, One-shape, and Neolix) regarding their ability to induce intracanal dentinal defects. Results indicated that all rotary systems were capable of causing dentinal defects. The highest dentinal crack rate occurred with the use of One-shape single rotary files, while the lowest rate was observed with One-curve rotary files.

We selected mandibular incisors for this study, consistent with previous research, as they are more prone to dentinal cracks due to their smaller proportions [ 4 , 6 ].

Notably, the absence of cracks in the negative control group in our study is consistent with literature findings, suggesting that the sectioning procedure does not produce dentinal defects [ 21 - 22 ].

In this current *ex vivo* study, the PDL was reconstructed using a light body silicone-based impression material, allowing the teeth to have limited movement and preventing external reinforcements [ 21 ]. The role of the periodontal ligament (PDL) in receiving and distributing stress and strain on teeth due to its viscoelastic characteristics is crucial. However, some studies have neglected to reconstruct the PDL in the methods of their study [ 3 , 21 ].

Onnink . [ 23 ] were the first to identify that canal preparation methods can lead to dentinal defects. Excessive cleaning and shaping can reduce intra-canal dentin thickness and weaken root structure. Additionally, rotary files generate different degrees of rotational forces on dentinal walls, potentially resulting in microcracks or craze lines [ 5 ]. We observed a 53% crack formation rate with One-Shape rotary files, higher than the 29% reported by Shantiaee . [ 24 ]. However, Pedullà . [ 25 ] reported a 75% crack formation rate with the One-shape system, although they did not establish a glide path in their study. These discrepancies may stem from differences in glide path establishment methods, as well as variations in speed and torque settings.

The reported percentage of crack formation varies in previous investigations [ 3 - 5 ] involving the use of ProTaper Universal. Our study found a 33% crack formation rate, whereas Liu . [ 4 ] reported 50% when using mandibular incisors. Additionally, Yoldas . [ 5 ] observed 30% crack formation in mesial roots of mandibular molars, and Bier . [ 3 ] reported 16% crack formation in mandibular premolars. These discrepancies are likely due to differences in the teeth studied.

Instrument design plays a role in dentinal defect formation [ 11 , 26 ]. The S-shape cross-sectional design of the One-shape rotary system, with its two cutting edges, may reduce the screwing effect and influence dentinal defects. Our study revealed a higher but not significant likelihood of dentinal defects with One-shape instruments, consistent with findings by Burklein . [ 11 ] and Gergi . [ 26 ], indicating that the S-shape design is more prone to creating defects than modified triangular or triangular cross-sections.

Similar to findings by Ozlek . [ 27 ], we observed fewer dentinal defects with the One-curve rotary system, likely due to its manufacturing design. The One-curve rotary file, made from a heat-treated Ni-Ti alloy (C-Wire), features shape memory, increased flexibility with a triangular cross-section, and variable shape designs [ 13 ]. In this study, Neolix rotary systems demonstrated 33% crack rates which was similar to Protaper rotary group. It is speculated that the manufacturing process of Neolix files, combined with their rectangular cross-section, provides them with higher flexibility, increased cutting efficiency, and reduced intra-canal stress. However, Priya . [ 28 ] found that Neolix single files induced more dentinal cracks compared to the ProTaper system, contrary to our results.

The influence of the number of instruments in a rotary system and instrument motion used for canal preparation on crack incidence remains a debated topic. Although we did not directly investigate the influence of these factors on crack formation, our findings align with previous research suggesting that rotary instrumentation can induce various dentinal defects [ 11 , 21 , 28 ]. Several studies [ 4 , 21 , 29 ] have reported significantly higher dentinal crack rates with continuous motion compared to reciprocating motion. However, some researchers have found higher crack rates with rotary systems utilizing reciprocating motion [ 11 , 26 ]. As mentioned earlier, there are a large number of discrepancies in the literature regarding the incidence of crack formation with different rotary files. Therefore, performing meta-analysis studies is suggested to shed light on this topic.

## Conclusion

Within the limitations of this study, we found that all experimented rotary systems resulted in the development of dentinal cracks. Although no significant difference was observed between the experimental groups, the fewer crack in one-curve group may make it a safer choice for clinical use. 
